# Case Report: Dilated cardiomyopathy as the initial presentation in an adult with late-onset CblC defect

**DOI:** 10.3389/fcvm.2025.1610295

**Published:** 2026-01-22

**Authors:** Dongling Xu, Chi Zhang, Lin Hao, Shaojie Bi, Aiying Xue, Liangshuai Yuan, Wenke Wang

**Affiliations:** Department of Cardiology, The Second Qilu Hospital of Shandong University, Jinan, Shandong, China

**Keywords:** dilated cardiomyopathy, heart failure, late-onset cblC, methylmalonic acidemia, renal dysfunction

## Abstract

Combined methylmalonic aciduria and homocystinuria, cobalamin C (cblC) type, represents the most common inborn error of cobalamin metabolism, caused by pathogenic variants in the *MMACHC* gene. We report the case of a 27-year-old Chinese woman who presented with dilated cardiomyopathy and renal insufficiency. Blood amino acid and acylcarnitine profiling revealed elevated ratios of propionylcarnitine (C3) to acetylcarnitine (C2) and C3 to free carnitine (C0). Genetic testing identified compound heterozygous pathogenic variants in *MMACHC*—*c.80A* *>* *G, p. (Gln27Arg)* and *c.609G* *>* *A, p. (Trp203Ter)*—confirming the diagnosis of cblC-type methylmalonic aciduria with homocystinuria. Despite administration of vitamin B12 and betaine, her heart function did not improve. The patient eventually succumbed to severe COVID-19 infection, which led to metabolic acidosis, renal failure, and multi-organ failure. This case underscores the challenging clinical course of late-onset cblC disorder and contributes to its expanding phenotypic spectrum.

## Introduction

Combined methylmalonic aciduria and homocystinuria, cobalamin C (cblC) type, represents the most common inborn error of intracellular cobalamin metabolism. CblC disease can manifest at any age, with clinical presentations varying widely, depending on the age of onset, ranging from prenatal to adulthood (1). However, late-onset cblC disease is less common than the early-onset form. The most frequent clinical manifestations are neurological symptoms, although renal injury, pulmonary arterial hypertension, ocular abnormalities, and thrombotic complications have also been reported (2, 3). In late-onset cases, cognitive impairment and psychiatric disturbances are more prominent (4). Cardiovascular involvement, including cardiomyopathy, congenital heart disease, and arrhythmias, is rarely reported in MMA (5, 6). Progressive cardiomyopathy and heart failure related to cblC have previously been documented only in early onset disease (7). To our knowledge, dilated cardiomyopathy leading to heart failure as the initial presentation in an adult with late-onset MMA has not been reported. We present such a case of late-onset cblC deficiency in an adult who first presented with dilated cardiomyopathy.

## Case presentation

A 27-year-old woman was hospitalized with a five-day history of progressive cough and chest tightness, which worsened in the supine position. She had no significant prior medical history, exhibited normal intelligence and vision, showed no evidence of maculopathy, and had no remarkable family history. She had previously given birth to a healthy boy before being diagnosed with cblC disease and had experienced one miscarriage approximately one year before this admission.

Physical examination revealed a blood pressure of 102/63 mmHg, a pulse of 98 beats/min, and no lower limb edema. Scattered wet rales were audible bilaterally. No cardiac murmurs or gallop rhythms were detected. Cranial nerve examination showed no abnormalities, and muscle strength and tone were normal in all four extremities.

Relevant laboratory tests showed normal liver function, electrolytes, glucose, thyroid function, coagulation profile, platelet count, and myocardial injury markers (Table 1). Brain natriuretic peptide (BNP) was markedly elevated at 1735 pg/mL (reference 0–100 pg/mL). The D-dimer level exceeded the upper limit of the reference range (556 ng/mL, reference: 0–500 ng/mL). Serum creatinine was elevated (118 μmol/L, reference: 41–73 μmol/L), and the estimated glomerular filtration rate (eGFR) was reduced (54.6 mL/min/1.73m^2^, reference: >60 mL/min/1.73m^2^). Urinalysis indicated proteinuria (++), and 24-h urine microalbumin level was elevated (124.08 mg, reference: 0–30 mg/24 h). Homocysteine (HCY) was elevated at 213.8 μmol/L (reference: 0–15 μmol/L). Vitamin B12 levels were also elevated (1,088 pg/mL, reference: 180.00–914.00 pg/mL). Urinary methylmalonic acid was markedly elevated (0.1141, reference:<0.001), representing a 114-fold increase. Plasma amino acid and acylcarnitine analysis revealed an elevated propionylcarnitine (C3) level (4.249 μM, reference: 0.00–3.58 μM). The ratios of C3 to acetylcarnitine (C2) (0.335, normal: 0.01–0.24) and C3 to free carnitine (C0) (0.308, reference: 0.04–0.15) were also increased. Plasma amino acid methionine level was normal (9.611 μM, reference: 2.80–25.3 μM).

**Table 1:** Laboratory tests of patient at first visit.

Test	Result	Reference value
AST	15	13–40 U/L
ALT	9	7–45 U/L
*γ*-GGT	14	7–45 U/L
ALP	47	35–135 U/L
GLU	4.56	3.90–6.10 mmol/L
LDH	232	120–250 U/L
Scr	118	41–73 μmol/L
CysC	1.57	0.55–1.20 mg/L
BUN	6.62	2.6–7.5 mmol/L
K	4.33	3.50–5.30 mmol/L
Na	137	137–147 mmol/L
Cl	104	99–110 mmol/L
Hs-cTnI	<0.01	0–0.03 ng/mL
BNP	1,735	0–100 pg/mL
FT3	5.52	3.53–7.37 pmol/L
FT4	13.17	7.98–16.02 pmol/L
TSH	1.65	0.560–5.910 pmol/L
PT	12.2	8.80–13.80 s
APTT	31.7	26.0–42.0 s
INR	1.09	0.80–1.20
FIB	2.85	2.00–4.00 ng/mL
PLT	221	125–350 × 10^9^/L

AST, aspartate aminotransferase; ALT, alanine aminotransferase; γ-GGT, γ-Glutamyl transpeptidase; ALP, alkaline phosphatase; GLU, glucose; LDH, lactate dehydrogenase; Scr, serum creatinine; CysC, cystatin C; BUN, blood urea nitrogen; Hs-cTnI, high-sensitivity cardiac troponin I; BNP, B-type natriuretic peptide; FT3, free triiodothyronine; FT4, free thyroxine; TSH, thyroid stimulating hormone; PT, prothrombin time; APTT, activated partial thromboplastin time; INR, international normalized ratio; FIB, fibrinogen; PLT, blood platelet.

**Figure 1:**
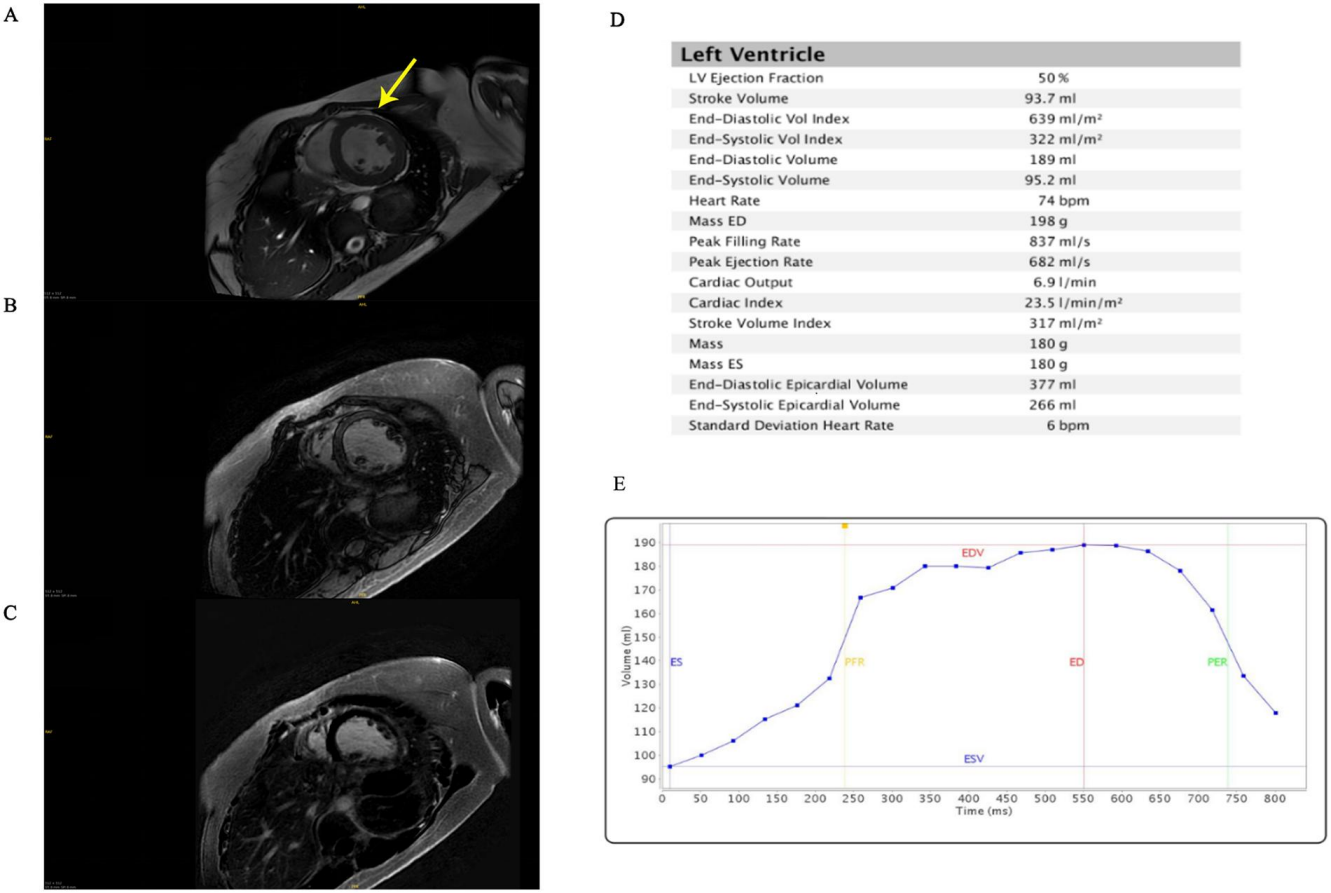
Cardiac magnetic resonance imaging. **(A)** T1 mapping. The arrow points to the presence of abundant muscle trabeculae in the left ventricle. **(B)** Late gadolinium enhancement. **(C)** T2 fat saturation. **(D)** Left ventricular parameters. **(E)** Time-volume function. ES, end-systolic; ED, end-diastolic; EDV, end-diastolic volume; ESV, end-systolic volume; PFR, peak filling rate; PER, peak rejection rate.

Echocardiography demonstrated a dilated left ventricle (left ventricular end diastolic diameter: 64 mm), left ventricular non-compaction, severe mitral regurgitation, mildly elevated pulmonary arterial systolic pressure (37 mmHg), and severely reduced left ventricular ejection fraction (34%). Cardiac magnetic resonance imaging confirmed left ventricular dilatation and prominent apical trabeculation (Figure 1). However, the findings did not meet the criteria for left ventricular non-compaction (LVNC) (8). Electrocardiography (ECG) showed no signs of myocardial ischemia.

Magnetic resonance imaging (MRI) of the brain was unremarkable. Renal ultrasound revealed increased cortical echogenicity and loss of corticomedullary differentiation. No evidence of pulmonary inflammation was found on chest computed tomography.

**Figure 2:**
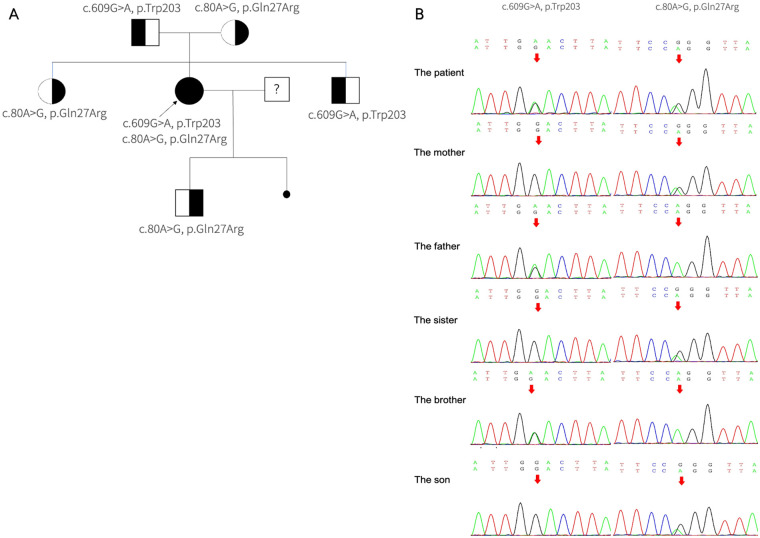
The pathogenic variants detected in MMACHC. **(A)** The pedigree of the family with MMA. The arrows suggest the proband, her parents, brother, sister, and son have no signs of MMA. She has one miscarried child with unknown genotype. **(B)** Segregation analysis of the pathogenic variants within the family revealed that the proband is a compound heterozygote. The *c.80A* *>* *G* mutation was maternally inherited, identified in her mother, sister, and son. Conversely, the *c.609G* *>* *A* mutation was paternally inherited, found only in her father and brother.

Genetic analysis identified compound heterozygous pathogenic variants in the MMACHC gene: *c.80A* *>* *G, p. (Gln27Arg) and c.609G* *>* A, p. (Trp203*) (Figure 2). Cascade molecular testing of the parents confirmed that the variants were in an “in trans” configuration in the proband and revealed carrier status in additional family members. The MMACHC gene pathogenic variants were *c.609G* *>* *A* in her father and *c.80A* *>* *G* in her mother (Figure 2). Her child carried the *c.80A* *>* *G* heterozygous pathogenic variants in MMACHC gene. Whole-exome sequencing did not detect any other known cardiomyopathy-associated pathogenic variants. These findings suggest that the patient's myocardial injury may be related to congenital metabolic disorder. The final diagnosis was late-onset cblC disease accompanied by dilated cardiomyopathy.

The patient was initially treated with hydroxocobalamin (1 mg/day) and betaine (9 g/day). Furosemide (20 mg/day) was administered to alleviate symptoms of heart failure. In addition, sacubitril/valsartan (50 mg/day) and metoprolol (23.75 mg/qd) were also prescribed as anti-myocardial remodeling drugs. Following treatment, the patient's dyspnea resolved and NT-proBNP levels returned to normal, leading to her discharge from the hospital.

After 1 month of treatment, her urine methylmalonate acid level decreased significantly to 0.052 (reference range:<0.001) but remained above normal. Accordingly, the hydroxocobalamin dose was increased to 10 mg daily. However, due to a limited understanding of her illness, financial constraints, fatigue from frequent medical appointments, and a fear of blood draws, the patient often avoided regular follow-up. As a result, amino acid and acylcarnitine profiling was not performed.

**Figure 3:**
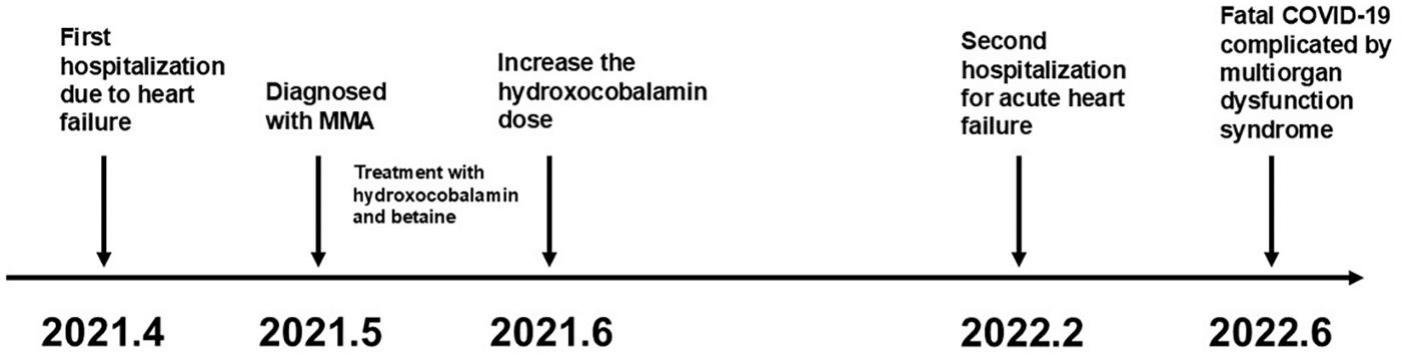
Timeline of disease progression.

Renal and heart functions remained stable but persistently impaired over more than one year of follow-up (Table 2)*.* Two years after the initial diagnosis, the patient contracted coronavirus disease 2,019 (COVID-19). Within two weeks, her cardiac and renal functions deteriorated rapidly, accompanied by significant elevations in D-dimer and HCY. She subsequently developed disseminated intravascular coagulation (DIC) and multiorgan failure, ultimately leading to unsuccessful resuscitation and death. The overall clinical course is summarized in Figure 3.

**Table 2:** Follow-up echocardiographic assessments and renal function tests.

	Renal function			Cardiac function		
Time	Creatinine (μmol/L)	BUN (mmol/L)	eGFR (*N*, 90–110 mL/min/1.73m^2^)	Ejection fraction (EF) (*N*, 50–60%)	Left ventricular end diastolic dimension (LVDd) (*N*, 35–50 mm)	Pulmonary artery systolic pressure (PASP) (*N*, 10–30 mmHg)
Admission	118	6.62	54.6	34%	64 mm	37
1-month follow-up	108	7.40	60.8	39%	67 mm	–
10-month follow-up	122	10.7	48.3	39%	66 mm	–

## Discussion

We reported the case of a 27-year-old woman with late-onset cblC disease manifesting as dilated cardiomyopathy and renal dysfunction. The diagnosis was confirmed through comprehensive metabolic and genetic investigations, which revealed markedly elevated homocysteine levels (213.8 μmol/L), significantly increased methylmalonic acid (114-fold above normal), and compound heterozygous pathogenic variants in the MMACHC gene (*c.80A* *>* *G* and *c.609G* *>* *A*).

Methylmalonic acidemia (MMA) is the most common organic aciduria in China, with the combined methylmalonic acidemia and homocystinuria subtype accounting for 60%–80% of cases. Among these, the cblC type is predominant, comprising approximately 95% of all cases (9). CblC disease is an autosomal recessive disorder caused by pathogenic variants in the MMACHC gene (located on chromosome *1p34.1*). Clinical manifestations vary significantly depending on the age of onset. Based on this factor, the disease is categorized into early-onset and late-onset types. Patients who develop overt symptoms after 4 years of age have been defined as late-onset type. Early-onset patients typically present more severe symptoms within the first year with multisystem disease, including neurological, ocular, renal, cardiac, and pulmonary manifestations. In contrast, late-onset patients generally exhibit a milder clinical phenotype, primarily affecting the central nervous system (10).

Given its heterogeneous and non-specific symptoms, late-onset cblC is often under-recognized, leading to frequent misdiagnosis or delayed diagnosis. This case represents the first documented instance of late-onset cblC disease presenting with cardiomyopathy as the primary manifestation. This finding significantly expands the known clinical spectrum of cblC disease and highlights the importance of considering metabolic disorders in the differential diagnosis of adults presenting with unexplained cardiac symptom.

Cardiac complications are infrequently reported in MMA (11), with late-onset cblC patients typically exhibiting neurological symptoms. Although cardiomyopathy has been documented in some cobalamin metabolism disorders, including cblC deficiency, such cases have predominantly occurred in early-onset disease. Our literature review indicates that cardiovascular involvement in late-onset cblC is exceptionally rare and, in all previously documented cases, was accompanied by neurological manifestations (Table 3) (5, 6, 12–22). Here, we present the first reported case of late-onset cblC disease initially manifesting with dyspnea as the primary symptom and isolated dilated cardiomyopathy, in the absence of neurological involvement. This atypical presentation highlights diagnostic challenges, as the absence of neurological symptoms and late onset frequently lead to missed diagnoses.

**Table 3:** Documented cases of patients with cblC defect who presented with cardiac disease.

NO (reference)	Report year	Age at diagn	Clinical Manifestations	Types of heart disease	serum HCY（µmol/L）	MMA（mmol/ mol cr）	MMACHC m	Treatment regimen	Outcome
1 (12)	1999	5 months	Congenital malformations	Pulmonic stenosis	↑	↑	N/A	CBL	Improved
2 (12)	1999	2 months	Congenital malformations	VSD	↑	↑	N/A	CBL	Improved
3 (13)	2001	3 weeks	Feeding difficulties, failure to thrive	VSD	282	1,914 (in urine)	N/A	CBL, F, CYS,PR	Improved
4 (14)	2009	Prenatal	Growth restriction	DCM, VSD	236	↑	N/A	CBL, PR, CAR, F, CYS	Improved
5 (6)	2009	birth	Found during routine screening	ASD, LVEF decrease	63	29 (in urine)	568insT/568	CBL, PR, CAR, F	Improved
6 (6)	2009	2 months	Found during routine screening	Mitral valve prolapse and Mild mi	95	266 (in urine)	271dupA/2	CBL, PR, CYS, F	Improved
7 (6)	2009	Prenatal	Found during routine screening	Focal LVNC	107	196 (in urine)	271dupA/2	CBL, PR, CYS, F	Improved
8 (6)	2009	3 years	Found during routine screening	Normal structure, LVEF decrease	99	57 (in urine)	C666A/C666	CBL, PR, CYS, ASA	Improved
9 (6)	2009	3 months	Found during routine screening	VSD	69	74 (in urine)	C481 T/C481	CBL, PR, CYS, ASA	Improved
10 (6)	2009	2 months	Found during routine screening	Normal structure, LVEF decrease	35	24 (in urine)	G609A/G60	CBL, PR, CYS, CAR, F	Improved
11 (6)	2009	Birth	Found during routine screening	Normal structure, LVEF decrease	32	35 (in urine)	271dupA/2	CBL, PR, CYS, F, M, C	Improved
12 (6)	2009	Birth	Found during routine screening	ASD, focal LVNC	64	31 (in urine)	547–8delGT	CBL, PR, CYS, CAR, F	Improved
13 (6)	2009	Birth	Found during routine screening	Normal structure, LVEF decrease	30	34 (in urine)	271dupA/2	CBL, PR, CAR, F	Improved
14 (6)	2009	Birth	Found during routine screening	Normal structure, LVEF decrease	42	20 (in urine)	G608A/G60	CBL, PR, CAR, F	Improved
15 (5)	2013	Prenatal	Found during routine prenatal ultrasound	LVNC	180	330 (in urine)	c.271dupA	CAR, CBL, F, CYS, PR, ASA	Improved
16 (15)	2013	2 years	Feeding difficulties, failure to thrive	PAH	66.9	9.9μmol/L (in serum)	c.271dupA/c.A389G	CYS,CBL,F	Died
17 (16)	2013	1.5 years	Tachydyspnea	PAH	N/A	N/A	c.276G.T/c.271dupA	CBL	Died
18 (16)	2013	2.5 years	Tachydyspnea	PAH	123	14424 nmol/L (in serum)	c.464G.A/c.464G.A	CBL	Died
19 (16)	2013	3 years	Fatigue	PAH	185	1,546 nmol/L (in serum)	c.276G.T/c.442_444delinsA	CBL	Died
20 (16)	2013	4 years	Fatigue	PAH	142	8,602 nmol/L (in serum)	c.276G.T/c.271dupA	CBL	N/A
21 (16)	2013	14 years	Fatigue	PAH	147	N/A	c.276G.A/c.14_24del11	CBL	N/A
22 (17)	2017	21 months	Shortness of breath and fever	PAH, mild tricuspid and pulmonary valve regurgitation	↑	0.218 mg/dL (in serum); 0.428 mg/dL (in urine)	c.80A > G(*p*	CBL, CAR, CYS	Improved
23 (17)	2017	4 years	Cough, dyspnea	PAH, moderate tricuspid regurgitation and mild pulmonary valve regurgitation	>50.0	0.294 mg/dL (in serum); 0.354 mg/dL (in urine)	N/A	CBL, CAR, CYS	Died
24 (17)	2017	7 years	Mild wet cough and shortness of breath	PAH	193.76	0.383 mg/dL (in serum); 0.1034 mg/dL (in urine)	c.80A > G(*p*	CBL, F, CAR, CYS	Improved
25 (18)	2018	2 years	Severe respiratory symptoms, palmoplantar edema	PAH	74	138 (in serum); 919 (in urine)	c.271dupA	CBL, CAR, CYS	Improved
26 (19)	2019	2 months	Pneumonia, anemia	Heart failure	290	178.24 (in urine)	c.80A > G; c	N/A	N/A
27 (20)	2021	3-months	Failure to thrive, hypotonia and pallor	DCM	148	3482 (in urine)	c.271dupA	CAR, CBL, F,CYS	Improved
28 (21)	2022	4 years	Pale complexion, brown urine, vomiting, and fatigue.	Coronary artery ectasia	216.7	43.05 (in urine)	c.80A > G, p.(Q27R) and c.609G > A (W203X)	CBL, CRE, CYS, F, ASA	Improved
29 (22)	2025	3 years	Vomiting, edema	Hypertension, left heart enlargement	31.7	53.9 (in urine)	c.80A > G, p. (Gln27Arg) and c.609G > A, p. (Trp203*)	CAR, CBL, F, PR	Died
30 (22)	2025	6 years	Vomiting, shortness of breath, edema	Hypertension, left heart enlargement, non-compaction of ventricular myocardium	212.9	20.5 (in urine)	c.80A > G, p. (Gln27Arg) and c.609G > A, p. (Trp203*)	CAR, CBL, F, PR	Improved
31 (22)	2025	12 years	Lethargy, cough	Hypertension, left heart enlargement	136.6	44.3 (in urine)	c.80A > G, p. (Gln27Arg) and c.609G > A, p. (Trp203*)	CAR, CBL, F, PR	Improved
32 (22)	2025	6 years	Recurrent pneumonia	Enlarged heart, pulmonary hypertension	136	64.7 (in urine)	c.80A > G, p. (Gln27Arg) and c.609G > A, p. (Trp203*)	CAR, CBL, F, PR	Improved

CblC, cobalamin C deficiency; ASD, atrial septal defect; VSD, ventricular septal defect; DCM, dilated cardiomyopathy; PAH, pulmonary arterial hypertension; LVNC, left ventricular non-compaction; VSD, ventricular septal defect; ↑, larger than the reference value; CAR, carnitine; CBL, hydroxocobalamin; F, folic acid/folate; CYS, cystadane/betaine; PR, protein restriction; ASA, aspirin; M, methioniine; CRE, creatine; N/A, not available.

To demonstrate the relationship between dilated cardiomyopathy and cblC, whole-exome sequencing for this patient was performed. The result revealed no related genes associated with cardiomyopathy in this patient. Therefore, the myocardial damage in this patient might be attributed to congenital metabolic disease. The pathological mechanisms of the impact of cblC disease on the cardiovascular system are not yet fully understood. Mmachc mouse models have been used to study the pathophysiology of combined methylmalonic acidemia, and these animals also present with cardiac abnormalities, displaying thin, hypertrabeculated ventricles and ventricular septum defects (23). In this case, the patient's genetic analysis revealed a compound heterozygous mutation in the MMACHC gene [*c.80A* *>* *G*, *p. (Gln27Arg)* from her mother and *c. 609G* *>* A, p. (Trp203*) from her father]. These are the most commonly reported pathogenic variants in cblC deficiency in China (19, 22). This patient also presented with renal dysfunction, characterized by abnormal renal ultrasound findings and proteinuria. This renal impairment was an asymptomatic laboratory and imaging finding, discovered concurrently with the cardiac symptoms. The *c.80A* *>* *G, p. (Gln27Arg)* variant observed in this case has also been associated with prominent renal complications in Chinese cblC patients, with several cases of late-onset thrombotic microangiopathy/hemolytic uremic syndrome (24, 25). However, in this patient, a renal biopsy was not performed, precluding confirmation of thrombotic microangiopathy.

In the literature review of cblC, early-onset cases frequently presented with life-threatening cardiac involvement but typically demonstrated significant improvement with prompt vitamin B12 administration. Most cblC patients responded favorably to treatment, with resolution of both clinical symptoms and biochemical abnormalities. Although late-onset cases are generally associated with better long-term outcomes, data on patients with cardiac involvement remain limited. In the present case of MMA, while an optimal treatment regimen led to rapid normalization of BNP and resolution of wheezing symptoms, it did not reverse the existing myocardial remodeling. This suggests that the treatment was effective in managing acute hemodynamic stress but did not alter the chronic structural adaptation of the heart. The myocardial damage appeared irreversible and ultimately led to a poor outcome. The patient died from COVID-19-induced DIC and subsequent multi-organ failure. COVID-19 is known to damage multiple organs, including the heart and kidneys, through a common pathway of systemic dermatitis thrombotic microvascular disease. Renal failure (anuria, acidosis) and heart failure exacerbate each other, rapidly leading to a fatal crisis. Delayed diagnosis likely contributed to the progressive deterioration of cardiac function. Earlier intervention might have prevented irreversible organ injury. Our report suggests that although cblC disease is treatable when diagnosed early, delayed recognition and management can lead to irreversible consequences and even death. The later the onset, the worse the prognosis. Therefore, timely diagnosis and effective treatment are crucial to alleviate clinical symptoms and reduce mortality.

Although rare, late-onset combined methylmalonic aciduria and homocystinuria (cblC type) should be considered a cause of unexplained heart failure in adolescents or adults with markedly elevated homocysteine levels. When unexplained heart disease is present in adolescents or adults, cardiologists should consider inborn errors of metabolism in the differential diagnosis. Urine organic acid analysis and genetic testing can confirm the diagnosis of cblC disease.

## Data Availability

The datasets presented in this study can be found in online repositories. The names of the repository/repositories and accession number(s) can be found in the article/Supplementary Material.
